# Testing for Human Immunodeficiency Virus Among Cancer Survivors Under Age 65 in the United States

**DOI:** 10.5888/pcd11.140274

**Published:** 2014-11-13

**Authors:** Jun Li, Trevor D. Thompson, Eric Tai, Guixiang Zhao, Alexandra M. Oster

**Affiliations:** Author Affiliations: Trevor D. Thompson, Eric Tai, Guixiang Zhao, Alexandra M. Oster, Centers for Disease Control and Prevention, Atlanta, Georgia.

## Abstract

**Introduction:**

Knowing the human immunodeficiency virus (HIV) serostatus of patients at the time of cancer diagnosis or cancer recurrence is prerequisite to coordinating HIV and cancer treatments and improving treatment outcomes. However, there are no published data about HIV testing among cancer survivors in the United States. We sought to provide estimates of the proportion of cancer survivors tested for HIV and to characterize factors associated with having had HIV testing.

**Methods:**

We used data from the 2009 Behavioral Risk Factor Surveillance System to calculate the proportion of cancer survivors under age 65 who had undergone HIV testing, by demographic and health-related factors and by state. Adjusted proportion estimates were calculated by multivariable logistic regression.

**Results:**

Only 41% of cancer survivors in the United States under the age of 65 reported ever having had an HIV test. The highest proportion of survivors tested was among patients aged 25 to 34 years (72.2%), non-Hispanic blacks (59.5%), and cervical cancer survivors (51.2%). The proportion tested was highest in the District of Columbia (68.3%) and lowest in Nebraska (24.1%). Multivariable analysis showed that factors associated with HIV testing included being non-Hispanic black or Hispanic, being younger, having higher education, not being married or living with a partner, not being disabled, and having medical cost concerns. Having an AIDS-related cancer was associated with HIV testing only among females.

**Conclusion:**

The proportions of HIV testing varied substantially by demographic and health-related factors and by state. Our study points to the need for public health interventions to promote HIV testing among cancer survivors.

## Introduction

In 2010, about 50,000 new human immunodeficiency virus (HIV) infections occurred in the United States, and more than 1.1 million people were living with HIV infection (1,2). About 16% of HIV infections go undetected (2). Since 2006, the Centers for Disease Control and Prevention (CDC) has recommended HIV testing as a routine part of medical care for all persons aged 13 to 64 years (3). Compared with the general population, people with HIV infection have increased cancer risks; Kaposi sarcoma, non-Hodgkin lymphoma, and cervical cancer are AIDS-related cancers (4,5). Because of co-infections with hepatitis B and C and Epstein-Barr virus, HIV-infected people also have elevated risks for non-AIDS–related cancers, such as liver cancer and Hodgkin disease (6).

Driven by improvements in early detection and treatment, cancer survival rates have increased substantially in the past 30 years (7). By 2007, nearly 12 million US cancer survivors were living, and the number is projected to reach about 18 million by 2022 (8,9). Given the high prevalence of both cancer and HIV infection and the natural linkage between the two, occurrence of HIV infection in cancer survivors is not rare. Patel et al reported that for HIV-infected persons, having AIDS-related cancers, a lower cluster of differentiation (CD4) cell count, and high viral load at cancer diagnosis were positively associated with poor 5-year survival (10). Undiagnosed HIV infection among cancer patients may contribute to poor cancer treatment outcomes and delay timely HIV care and treatment, resulting in increased risks for death (11). Identification of cancer survivors who have never been tested for HIV infection can reduce that risk.

To our knowledge, little is known about HIV testing among cancer survivors (11). This study’s objective was to describe the proportion of cancer survivors tested for HIV by demographic and health-related variables, to provide state-specific estimates of HIV testing, and to characterize factors associated with HIV testing.

## Methods

### Data source

Data for this analysis came from the 2009 Behavioral Risk Factor Surveillance System (BRFSS), an ongoing, state-based, random-digit–dialed telephone survey of the noninstitutionalized US civilian population aged over 18 years. The BRFSS (www.cdc.gov/BRFSS/) collects information on diseases, health-related behaviors, preventive health practices, and access to health care in the United States. In 2009, BRFSS was conducted in all 50 states, the District of Columbia, Puerto Rico, the US Virgin Islands, and Guam. The median response rate for the 2009 BRFSS was 52.5%, and the median cooperation rate was 75% (12).

### Study subjects and variables

In accordance with CDC’s recommended HIV testing criteria (3), we restricted our analysis to persons who reported a history of cancer, were aged 18 to 64 years, and fell within the age range of the BRFSS survey.

### Cancer-related variables

Respondents were asked whether they had ever been told by a doctor, nurse, or other health care professional that they had cancer. Respondents who answered yes were asked how many different types of cancer they had had, the age at which they were first told that they had cancer, and for respondents who reported having had more than one type of cancer, which type of cancer had most recently been diagnosed. Respondents who were unsure about their history of cancer or who refused to answer the question were excluded from the analysis.

For cancer survivors, the duration of cancer survivorship was calculated by using the respondent’s current age and the age at first cancer diagnosis. Cancer types were grouped as AIDS-related cancers (non-Hodgkin lymphoma and cervical cancer) and non-AIDS-related cancers (ie, prostate, female breast, colon, and liver; melanoma and other skin cancers; and other non-AIDS–related cancers) (5). Information on Kaposi sarcoma was not available from the BRFSS questionnaires, so we were unable to include it among AIDS-related cancers.

### HIV-related variables

Respondents were also asked whether they had ever been tested for HIV. Persons were considered to have recent HIV risk behaviors if they answered yes to any of the following questions regarding the past year: 1) Have you used intravenous drugs? 2) Have you been treated for a sexually transmitted disease? 3) Have you given or received money or drugs in exchange for sex? and 4) Have you had anal sex without a condom?

### Other variables

We examined the following factors, which were potentially related to HIV testing: demographics (sex, race/ethnicity, age at interview, education, and marital status), psychological status (emotional support and life satisfaction), health care access (insurance status, being concerned about medical costs in the past 12 months, and having a routine checkup in the past 12 months), and disability status, which was defined as being limited in any way in any activities because of physical, mental, or emotional problems.

### Statistical analyses

We examined HIV testing among cancer survivors by stratifying by the aforementioned variables. Proportions tested for HIV were age-standardized to the age distribution of cancer survivors in the 2009 BRFSS. Ninety-five percent confidence intervals around the estimated proportions were calculated on the basis of a logit transformation. Statistical testing for differences in age-standardized measures was performed using general linear contrasts. We also calculated estimated proportions tested for each of the 50 states and the District of Columbia. A multivariable logistic regression model was fitted to determine the adjusted relationships between HIV testing and the aforementioned demographic and health-related variables among cancer survivors in the United States. Age and time since cancer diagnosis were treated as continuous variables in the model and transformed by using restricted cubic spline functions to allow for nonlinearity. The adjusted associations between discrete variables and HIV testing are presented as predictive margins. The predictive margin for a specific group represents the average predicted response if everyone in the sample had been in that group. Because AIDS-related cancer types vary for men and women, we included a term describing sex by AIDS-related cancer interaction in the model to assess whether the effect varied by sex. Statistical testing for the age-adjusted and multivariable analyses was based on the Wald *F* test. We performed the analysis by using SAS version 9.3 (SAS Institute Inc) with SUDAAN version 11.0.0 (RTI International) along with the RMS version 3.6–3 and survey packages version 3.28–2, components of R version 3.0.1 (R Foundation for Statistical Computing) to account for the complex sampling design and to allow for weighted estimates. Significance was set at *P* < .05.

## Results

Of 407,402 persons interviewed during 2009 who answered the questions about cancer history, 59,173 reported a history of cancer. Of these, 24,485 were aged 18 to 64 years and were included in our analysis. Most cancer survivors were women (62.3%), non-Hispanic white (83.5%), aged 45 to 64 years (74.6%), and had health insurance (89.5%). About 13% of cancer survivors had AIDS-related cancers: 11.5% had cervical cancer and 1.9% non-Hodgkin lymphoma. Approximately 3% of cancer survivors reported at least 1 HIV risk behavior in the past year.

Of cancer survivors aged 18 to 64 years, 40.8% reported having ever had an HIV test (Table 1). The proportion who reported HIV testing varied significantly by race/ethnicity, age, education, cancer type, time since cancer diagnosis, and HIV risk behaviors. Within these respective categories, those most likely to have been tested included non-Hispanic blacks (59.5%), patients aged 25 to 34 years (72.2%), patients with less than a high school education (45.0%), cervical cancer survivors (51.2%), survivors 5 to 9 years post cancer diagnosis (42.9%), and those who reported engaging in at least 1 HIV risk behavior in the past year (58.0%). Measures of emotional support, life satisfaction, employment status, marital status, disability status, health insurance, and medical cost concern also were significantly associated with HIV testing. History of HIV testing varied significantly by state, ranging from 68.3% in the District of Columbia to 24.1% in Nebraska (Table 2). Among all states, the District of Columbia (68.3), Maryland (50.6%) and California (48.7%) had the highest proportions of cancer survivors who reported having been tested for HIV.

**Table 1:** Age-Standardized^a^ Proportion of US Cancer Survivors Aged 18 to 64 Years^b^ Tested for HIV, Behavioral Risk Factor Surveillance System, 2009

Characteristics	N^c^	% (95% CI)	*P* Value	
**Total**	23,626	40.8 (39.7–42.0)		NA
**Sex**				
Male	7,180	41.6 (39.4–43.8)		.21
Female	16,446	40.0 (38.7–41.3)		
**Race/ethnicity**				
Non-Hispanic white	20,605	37.7 (36.6–38.9)		.001
Non-Hispanic black	1,207	59.5 (54.7–64.1)		
Hispanic	839	52.3 (46.9–57.7)		
Non-Hispanic other	764	48.3 (40.6–56.1)		
**Age, y**				
18–24	148	52.4 (39.6–64.8)		<.001
25–34	874	72.2 (66.7–77.1)		
35–44	2,535	59.1 (55.7–62.4)		
45–54	6,979	42.9 (40.8–45.0)		
55–64	13,090	26.2 (24.9–27.6)		
**Education**				
<High school graduate	1,382	45.0 (40.6–49.4)		<.001
High school graduate	5,879	35.8 (33.6–38.0)		
Some college	6,971	43.9 (41.7–46.2)		
College graduate	9,374	40.8 (39.0–42.6)		
**Employment**				
Employed	13,696	38.4 (37.0–39.9)		<.001
Not employed	9,886	44.9 (43.1–46.7)		
**Marital status**				
Married or living with a partner	14,858	36.4 (35.1–37.8)		<.001
Divorced, separated, or widowed	6,547	53.6 (51.3–55.8)		
Never married	2,150	54.7 (50.8–58.5)		
**Type of cancer**				
**AIDS-related cancer**				
Cervical cancer	2,600	51.2 (47.9–54.5)		<.001
Non-Hodgkin lymphoma	284	44.8 (34.7–55.4)		
**Non-AIDS–related cancer**				
Prostate	1,073	47.3 (42.7–51.9)		<.001
Female breast	3,771	37.3 (34.6–40.1)		
Colon	832	36.6 (30.7–42.9)		
Melanoma	2,407	35.0 (31.8–38.5)		
Other skin	5,357	36.7 (34.5–39.0)		
Liver	83	61.9 (48.3–73.9)		
Other	6,549	42.7 (40.5–44.9)		
**Time since cancer diagnosis, y**				
0–4	8,000	38.7 (36.8–40.6)		.04
5–9	5,084	42.9 (40.4–45.4)		
10–19	5,537	41.5 (39.3–43.8)		
≥20	4,473	39.7 (36.3–43.1)		
**Emotional support**				
Always	18,369	39.1 (37.8–40.4)		<.001
Sometimes	3,197	47.3 (44.2–50.4)		
Rare or none	1,909	47.0 (43.3–50.8)		
**Life satisfaction**				
Very satisfied	10,125	36.9 (35.2–38.6)		<.001
Satisfied	11,339	42.0 (40.4–43.7)		
Dissatisfied	1,531	52.1 (48.1–56.0)		
Very dissatisfied	479	53.1 (46.2–59.8)		
**Health insurance**				
Yes	21,239	40.4 (39.2–41.7)		.03
No	2,363	44.4 (41.1–47.7)		
**Time since last checkup**				
Within past year	17,848	41.3 (39.9–42.6)		.24
1 – <2 y	2,681	38.7 (35.6–41.9)		
2 – <5 y	1,421	40.8 (36.7–45.1)		
≥5 y	1,320	37.9 (33.8–42.2)		
Never	166	48.5 (36.8–60.3)		
**Medical cost concern**				
Yes	3,713	48.5 (45.8–51.2)		<.001
No	19,875	39.2 (37.9–40.5)		
**Ever had disability**				
Yes	8,225	49.9 (48.0–51.9)		<.001
No	15,306	36.4 (35.1–37.8)		
**HIV risk behaviors**				
Yes	520	58.0 (51.0–64.7)		<.001
No	23,069	40.5 (39.3–41.7)		

Abbreviation: HIV, human immunodeficiency virus; CI, confidence interval; NA, not applicable.

a Results for all variables except age were age-standardized to the age distribution of cancer survivors in the 2009 BRFSS.

b 612 participants were excluded because of missing HIV status.

c Numbers may not add up to totals because of “don't know,” “refused,” or missing responses.

**Table 2:** Age-Standardized^a^ Proportion of HIV Tests Among Cancer Survivors Aged 18 to 64 Years, by State, Behavioral Risk Factor Surveillance System, 2009

State	N	% (95% CI)
Total	23,626	40.8 (39.7–42.0)
Alabama	382	44.6 (37.9–51.4)
Alaska	131	45.1 (35.3–55.4)
Arizona	327	38.8 (31.2–46.9)
Arkansas	210	37.4 (30.4–44.9)
California	794	48.7 (44.6–52.8)
Colorado	679	42.1 (37.5–46.7)
Connecticut	322	38.3 (32.2–44.8)
Delaware	240	44.7 (37.9–51.7)
District of Columbia	189	68.3 (61.3–74.6)
Florida	762	46.5 (41.3–51.6)
Georgia	342	40.5 (34.7–46.5)
Hawaii	361	36.7 (30.4–43.5)
Idaho	330	30.0 (24.8–35.8)
Illinois	286	34.4 (27.7–41.8)
Indiana	525	39.5 (34.3–45.0)
Iowa	296	30.7 (24.1–38.2)
Kansas	1,064	34.2 (31.1–37.3)
Kentucky	629	36.2 (30.4–42.3)
Louisiana	463	46.4 (40.8–52.0)
Maine	501	38.1 (33.4–43.1)
Maryland	512	50.6 (45.2–56.0)
Massachusetts	896	42.2 (37.8–46.8)
Michigan	479	37.4 (32.0–43.1)
Minnesota	279	35.4 (29.5–41.7)
Mississippi	552	39.8 (35.1–44.6)
Missouri	282	31.1 (24.5–38.6)
Montana	465	41.5 (36.1–47.2)
Nebraska	779	24.1 (19.6–29.2)
Nevada	255	45.6 (36.7–54.7)
New Hampshire	352	37.4 (32.4–42.7)
New Jersey	644	39.2 (34.6–44.0)
New Mexico	502	43.7 (38.5–49.1)
New York	381	40.4 (34.4–46.7)
North Carolina	745	45.7 (41.4–50.1)
North Dakota	232	29.6 (23.6–36.4)
Ohio	525	30.0 (25.2–35.3)
Oklahoma	466	38.8 (34.3–43.5)
Oregon	306	40.5 (35.0–46.3)
Pennsylvania	475	34.2 (28.9–39.9)
Rhode Island	362	31.6 (26.2–37.6)
South Carolina	586	37.5 (32.1–43.3)
South Dakota	343	25.1 (19.6–31.4)
Tennessee	284	45.7 (38.6–53.0)
Texas	609	46.7 (40.6–52.8)
Utah	537	29.1 (24.7–33.9)
Vermont	417	39.7 (34.7–45.0)
Virginia	286	47.9 (41.5–54.4)
Washington	1,317	44.3 (41.0–47.7)
West Virginia	271	27.9 (22.8–33.5)
Wisconsin	250	29.2 (22.3–37.2)
Wyoming	404	38.0 (32.7–43.5)

Abbreviation: HIV, Human immunodeficiency virus; CI, confidence interval.

a Age-standardized to the age distribution of cancer survivors in the Behavioral Risk Factor Surveillance System, 2009.

**Figure 1:**
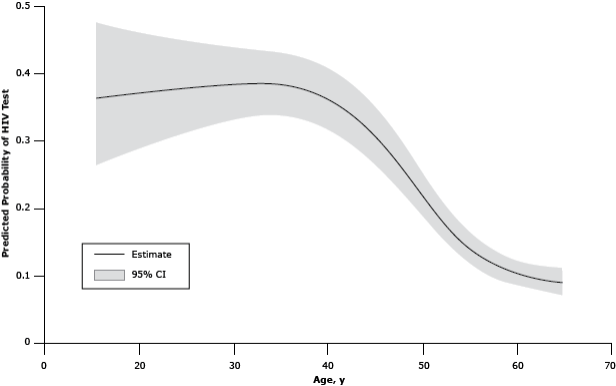
Model-adjusted relationship between age and human immunodeficiency virus (HIV) testing among cancer survivors aged 18 to 64 years, United States, 2009, Behavioral Risk Factor Surveillance System. Abbreviation: CI, confidence interval. **Table:** 

**Figure 2:**
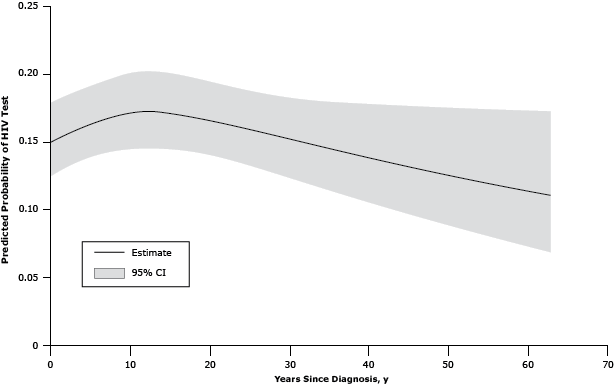
Model-adjusted relationship between age and human immunodeficiency virus (HIV) testing among cancer survivors aged 18 to 64 years, United States, 2009, Behavioral Risk Factor Surveillance System. Abbreviation: CI, confidence interval. **Table:** 

The multivariable logistic regression analysis (Table 3) revealed that women with an AIDS-related cancer, persons who had ever had a disability, and patients with medical cost concern were more likely to report having had an HIV test than men with cancer and women with non-AIDS–related cancer, those who did not have a disability or those who did not have medical cost concerns. Other significant findings included higher prevalences of testing among non-Hispanic blacks and Hispanics, patients with at least some college education, and patients who were not currently married or living with a partner. After adjusting for all variables, health insurance, emotional support, life satisfaction, employment status, and HIV risk behaviors were no longer significantly associated with HIV testing. There was a significant nonlinear association between age and HIV testing. Increasing age was significantly associated with a decline in HIV testing above age 35 (Figure 1). HIV testing prevalence tended to be slightly higher with increasing time since diagnosis up to around 10 years with slightly lower testing with increasing years since diagnosis beyond 20 years (Figure 2). However, this relationship was not significant (*P* = .07).

**Table 3:** Model-Adjusted Percentage of HIV Testing by Demographic and Health-Related Characteristics Among US Cancer Survivors Aged 18 to 64 Years, Behavioral Risk Factor Surveillance System, 2009

Characteristics	% (95% CI)	*P* Value^a^
**Sex^b^**	NA	<.001
**Type of cancer^b^**		
Male, AIDS-related cancer	34.2 (16.6–57.5)	<.001
Male, Non-AIDS–related cancer	44.4 (42.2–46.6)	
Female, AIDS-related cancer	48.8 (45.4–52.1)	
Female, Non-AIDS–related cancer	36.2 (34.6–37.7)	
**Race/ethnicity**		
Non-Hispanic white	38.3 (37.0–39.5)	<.001
Non-Hispanic black	55.5 (49.9–60.9)	
Hispanic	53.3 (46.6–59.9)	
Non-Hispanic other	45.9 (37.7–54.4)	
**Age, y**	Nonlinear	<.001
**Education**		
<High school graduate	36.9 (32.6–41.4)	<.001
High school graduate	33.3 (31.1–35.7)	
Some college	43.1 (40.8–45.3)	
College graduate	43.6 (41.7–45.5)	
**Employment**		
Employed	39.9 (38.3–41.5)	.18
Not employed	41.7 (39.7–43.7)	
**Marital status**		
Married or living together	37.4 (35.9–38.9)	<.001
Divorced, separated, or widowed	50.6 (48.2–53.0)	
Never married	46.2 (41.7–50.7)	
**Time since cancer diagnosis, y**	Nonlinear	.07
**Emotional support**		
Always	40.5 (39.1–41.9)	.23
Sometime	42.6 (39.1–46.1)	
Rare or none	38.1 (34.0–42.5)	
**Life satisfaction**		
Very satisfied	40.0 (38.0–42.0)	.31
Satisfied	40.5 (38.8–42.3)	
Dissatisfied	44.1 (39.5–48.7)	
Very dissatisfied	45.6 (37.8–53.6)	
**Health insurance**		
Yes	40.7 (39.4–42.1)	.63
No	39.7 (36.0–43.6)	
**Time since last checkup**		
Within past year	41.4 (40.0–42.8)	.20
1 – <2 y	38.2 (35.0–41.5)	
2 – <5 y	39.1 (34.8–43.6)	
≥5 y	36.8 (31.5–42.4)	
Never	44.5 (33.4–56.3)	
**Medical cost concern**		
Yes	44.5 (41.4–47.8)	.007
No	39.8 (38.4–41.2)	
**Ever had disability**		
Yes	47.6 (45.3–49.9)	<.001
No	37.2 (35.7–38.8)	
**HIV risk behaviors**		
Yes	47.3 (39.7–55.0)	.08
No	40.4 (39.1–41.7)	

Abbreviation: HIV, human immunodeficiency virus; CI, confidence interval; NA, not applicable.

a *P* value was calculated by using the Wald *F* test.

b Sex by type of cancer interaction term is included in the model (*P* = .06). *P* values are calculated from the simultaneous test so that the main effect and interaction coefficients are both equal to zero.

Age, y	Predicted Probability of HIV Test (95% CI)
18	0.36 (0.26–0.48)
19	0.37 (0.27–0.47)
20	0.37 (0.27–0.47)
21	0.37 (0.28–0.47)
22	0.37 (0.29–0.46)
23	0.37 (0.29–0.46)
24	0.37 (0.30–0.46)
25	0.37 (0.30–0.45)
26	0.38 (0.31–0.45)
27	0.38 (0.31–0.45)
28	0.38 (0.32–0.45)
29	0.38 (0.32–0.44)
30	0.38 (0.33–0.44)
31	0.38 (0.33–0.44)
32	0.39 (0.33–0.44)
33	0.39 (0.34–0.44)
34	0.39 (0.34–0.44)
35	0.39 (0.34–0.44)
36	0.38 (0.34–0.43)
37	0.38 (0.34–0.43)
38	0.38 (0.33–0.43)
39	0.37 (0.33–0.42)
40	0.37 (0.32–0.41)
41	0.36 (0.32–0.41)
42	0.35 (0.31–0.40)
43	0.34 (0.30–0.39)
44	0.33 (0.29–0.37)
45	0.31 (0.27–0.36)
46	0.30 (0.26–0.34)
47	0.28 (0.24–0.32)
48	0.26 (0.23–0.32)
49	0.24 (0.21–0.28)
50	0.22 (0.19–0.26)
51	0.20 (0.18–0.24)
52	0.18 (0.16–0.21)
53	0.17 (0.14–0.20)
54	0.15 (0.13–0.18)
55	0.14 (0.12–0.16)
56	0.13 (0.11–0.15)
57	0.12 (0.10–0.14)
58	0.11 (0.09–0.13)
59	0.11 (0.09–0.13)
60	0.10 (0.09–0.12)
61	0.10 (0.08–0.12)
62	0.10 (0.08–0.12)
63	0.09 (0.08–0.11)
64	0.09 (0.07–0.11)

Age, y	Predicted Probability of HIV Test (95% CI)
0	0.15 (0.12–0.18)
1	0.15 (0.13–0.18)
2	0.16 (0.13–0.18)
3	0.16 (0.13–0.19)
4	0.16 (0.14–0.19)
5	0.16 (0.14–0.19)
6	0.17 (0.14–0.19)
7	0.17 (0.14–0.20)
8	0.17 (0.14–0.20)
9	0.17 (0.14–0.20)
10	0.17 (0.15–0.20)
11	0.17 (0.15–0.20)
12	0.17 (0.15–0.20)
13	0.17 (0.15–0.20)
14	0.17 (0.15–0.20)
15	0.17 (0.15–0.20)
16	0.17 (0.14–0.20)
17	0.17 (0.14–0.20)
18	0.17 (0.14–0.20)
19	0.17 (0.14–0.20)
20	0.17 (0.14–0.20)
21	0.17 (0.14–0.19)
22	0.16 (0.14–0.19)
23	0.16 (0.14–0.19)
24	0.16 (0.14–0.19)
25	0.16 (0.13–0.19)
26	0.16 (0.13–0.19)
27	0.16 (0.13–0.19)
28	0.16 (0.13–0.19)
29	0.15 (0.13–0.18)
30	0.15 (0.13–0.18)
31	0.15 (0.12–0.18)
32	0.15 (0.12–0.18)
33	0.15 (0.12–0.18)
34	0.15 (0.12–0.18)
35	0.15 (0.12–0.18)
36	0.14 (0.11–0.18)
37	0.14 (0.11–0.18)
38	0.14 (0.11–0.18)
39	0.14 (0.11–0.18)
40	0.14 (0.11–0.18)
41	0.14 (0.10–0.18)
42	0.14 (0.10–0.18)
43	0.13 (0.10–0.18)
44	0.13 (0.10–0.18)
45	0.13 (0.10–0.18)
46	0.13 (0.10–0.18)
47	0.13 (0.09–0.18)
48	0.13 (0.09–0.18)
49	0.13 (0.09–0.18)
50	0.13 (0.09–0.18)
51	0.12 (0.09–0.17)
53	0.12 (0.08–0.17)
54	0.12 (0.08–0.17)
55	0.12 (0.08–0.17)
56	0.12 (0.08–0.17)
57	0.12 (0.08–0.17)
58	0.12 (0.08–0.17)
59	0.12 (0.08–0.17)
62	0.11 (0.07–0.17)
63	0.11 (0.07–0.17)

## Discussion

Our study, based on the largest telephone survey of adults in the United States, found that only 41% of US cancer survivors under age 65 in 2009 had ever had an HIV test. The likelihood of having had HIV testing varied markedly by state. Factors associated with HIV testing included being non-Hispanic black or Hispanic, younger age, having higher education, not being married or living with a partner, being disabled, and having medical cost concerns. Having an AIDS-related cancer was associated with HIV testing only among women.

Although information on the proportion tested for HIV is lacking for cancer survivors, it has been widely reported for the US general population. In 2013, CDC reported, on the basis of the National Health Interview Survey (NHIS), that the percentage of adults who had ever been tested for HIV significantly increased from 36.6% in 2000 to 45.0% in 2010 (13). Similar to what we have observed among cancer survivors, NHIS analyses showed that among the general population, non-Hispanic blacks, people aged 25 to 34 years, and people with a reported risk for HIV infection had the highest proportion of HIV testing (13). A British study examined HIV testing among cancer patients in a clinic where HIV testing was recommended for all lymphoma patients (14). This study reported that only 59% of 113 lymphoma patients underwent an HIV test after their cancer diagnosis, and a high prevalence of HIV co-infection (8.0%) was observed (14). In 2013, the US Preventive Services Task Force also recommended that clinicians screen adolescents and adults aged 15 to 65 years who were at average risk for HIV infection (15). The low percentage of HIV testing reported by our study emphasizes the need for more efforts to promote HIV testing among cancer survivors. Our study may serve as a baseline measure to monitor changes in HIV testing among cancer survivors.

Compared with the overall population, adolescents and young adults are disproportionately affected by HIV (3). This finding may partially account for the decline in HIV testing with age, as suggested by our study. However, among persons living with a diagnosed HIV infection, the percentage aged 50 years or older increased from 28.6% in 2007 to 32.7% in 2009 (1). One possible explanation is that HIV testing increased over time among this older population (13). The positive associations between HIV testing and disabilities as identified in our study were consistent with a 2002 NHIS study among the general population (16). That study revealed that adults with physical and mental disability were more likely to report having had an HIV test than nondisabled adults (16). One explanation for the greater likelihood of HIV testing among physically and mentally disabled adults was their increased risk of rape and sexual abuse and a higher rate of participation in HIV risk behaviors (16).

Our study shows that about 60% of US cancer survivors under age 65 have never had an HIV test. Although non-Hodgkin lymphoma and cervical cancers are AIDS-related cancers, almost half of patients with these cancers had never been tested for HIV. The nontargeted “opt-out” HIV testing (the test will be performed unless the patient declines) in all health care settings, recommended by CDC in 2006, has not been widely implemented (11). This may, in part, contribute to the low reports of HIV testing among cancer survivors. Other contributing factors have not been reported in the literature. However, studies have shown that for the general population, fear, stigma, and discrimination associated with an HIV diagnosis; physicians’ attitudes; and the need for additional health care personnel and effort affect adoption of HIV testing (13,17,18). In addition, the cost or reimbursement of the cost for HIV testing are shared concerns of both patients and health care providers (19). In our study, we found that only 16.7% of cancer survivors reported having medical cost concerns, and cancer survivors who had medical cost concerns were more likely to have had an HIV test than cancer survivors who had no such concerns. In 2009, the Centers for Medicare and Medicaid Services allowed reimbursement for routine HIV testing (20). However, the changes in Medicare and Medicaid coverage may not have had a substantial effect on HIV testing in our study population because of the age limits of 18 to 64 years and the time the survey was conducted. One possible explanation is that medical cost concerns are a surrogate for having low income. Those who have low income may be perceived to be at higher risk for HIV by health care providers and, therefore, may be more likely to be tested. Additional research is warranted to further investigate this issue.

Separate written consent for HIV testing among nonpregnant adults is another well recognized barrier. Twenty states had laws or regulations that required separate written consent when CDC released its 2006 HIV testing recommendation (17). By 2008, 11 states, including California and Maryland, enacted new legislation or regulations to remove this barrier (17). Our findings that in 2009, the District of Columbia, Maryland, and California had the highest reports of HIV testing among all the states may be partially due to the absence of requirements for separate consent in these states. Removal of requirements for a separate written consent for HIV testing in every state would likely promote HIV testing and reduce variations among states.

Kaposi sarcoma, non-Hodgkin lymphoma, and cervical cancer occur in excess in persons living with HIV (5). These cancers have been deemed AIDS-related conditions (5). Previous studies also indicate that HIV-infected persons are at higher risk than the general population for several non-AIDS–related cancers, such as anal cancer, Hodgkin disease, liver cancer, lung cancer, melanoma, and colorectal cancer (21,22). Besides the advent of highly active antiretroviral therapy, which has prolonged survival of HIV-infected patients, other factors that may account for increased cancer risk are immunosuppression (23,24), co-infection with an oncogenic virus (25–27), and unhealthy behaviors such as smoking (28). Awareness of HIV status is important at the time of cancer diagnosis or cancer recurrence to avoid treatment-related complications from co-infection with HIV, drug interactions, potential effects of chemotherapy on the CD4 cell count, and HIV viral load (14). Early identification of patients with HIV infection would allow early implementation of HIV infection prevention and control measures to reduce hospital-acquired infections among patients and health care providers (29,30). Coordination of anticancer and anti-HIV therapies could improve treatment outcomes among HIV-infected adults newly diagnosed with cancer or with cancer recurrence. Additionally, by knowing patients’ HIV status, health care providers can identify cancer survivors who are at elevated risk for a secondary cancer and educate them about the need for cancer screenings.

Our study has several limitations. First, BRFSS data were self-reported and subject to recall bias. Recall bias may be responsible for the slightly higher prevalence of certain cancers when compared with a study that used cancer registry data (8). HIV testing history data were self-reported and may be subject to social desirability or recall biases. Opt-out testing may also contribute to recall biases. For instance, patients may not have explicitly consented, so they may have been unaware when they were tested. Because of the limitations of recall bias and self-report, this study may seriously underestimate actual HIV testing among cancer survivors. Second, we are not able to determine whether the testing occurred before, at the time of, or after their cancer diagnosis. Third, because the 2009 BRFSS sampled only respondents with a landline telephone, persons without a landline telephone were excluded from this study. Fourth, BRFSS is limited to noninstitutionalized US citizens; cancer survivors who lived in nursing homes, long-term care facilities, or hospice were excluded. Last, the low response rate of 52.2% in the 2009 survey may limit the generalizability of the results to all cancer survivors living in the United States.

In conclusion, in 2009 about 60% of US cancer survivors under age 65 had never had an HIV test. The proportion of HIV testing varied substantially by demographic and health-related factors and by state. Increasing the proportion of new HIV infections diagnosed before progression to AIDS is a Healthy People 2020 (www.healthypeople.gov/2020/topicsobjectives2020/default) objective. One of the targets in the National HIV/AIDS Strategy is to increase the percentage of people who are living with HIV and know their serostatus from 79% to 90% by 2015. (13) Knowing the HIV serostatus at the time of cancer diagnosis or cancer recurrence is prerequisite to coordinating HIV and cancer treatments and improving treatment outcomes. Thus, our study points to the need for public health interventions to promote HIV testing among cancer survivors, especially among the demographic subgroups and states with a low proportion of HIV testing. With HIV testing, health care providers also can identify cancer survivors who are at elevated risk for a secondary cancer and educate them about the need for cancer screenings.
